# Supplementary material for: Electronic structure of quasi-one-dimensional superconductor K2Cr3As3 from first-principles calculations
Source: Sci Rep. 2015 Nov 3;5:16054. doi: 10.1038/srep16054 (PMC4630611; doi:10.1038/srep16054)
Supplement: Supplementary Information [file srep16054-s1.pdf]

Supplementary Information for Electronic structure of quasi-one-dimensional superconductor $K_2Cr_3As_3$ from first-principles calculations

by Hao Jiang, Guanghan Cao and Chao Cao

Relativistic DOS and PDOS of $K_2Cr_3As_3$ with experimental structure

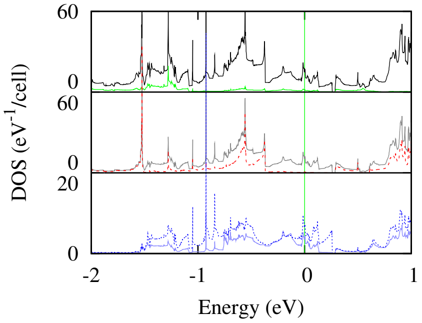

FIG. S-1: Total and projected DOS from relativistic calculations. The upper panel shows the total DOS (black line) and PDOS from As-4p (green line); middle and bottom panel shows the PDOS from all five-orbitals of Cr-3d (grey lines in the middle panel), the PDOS of Cr-3d$_{zx}$ and Cr-3d$_{zy}$ (red line in the middle panel), and the PDOS of Cr-3d$_{z^2}$ (light blue line in the bottom panel), Cr-3d$_{x^2-y^2}$ and Cr-3d$_{xy}$ (blue line in the bottom panel).

We show here in FIG. S-1a the DOS and PDOS of $K_2Cr_3As_3$ from relativistic calculations (with SOC effect included). The lattice constants and internal coordinates from experiment were employed. The orbital contribution close to E_F remains the same as non-relativistic calculations.

Electronic structure with fully optimized structure

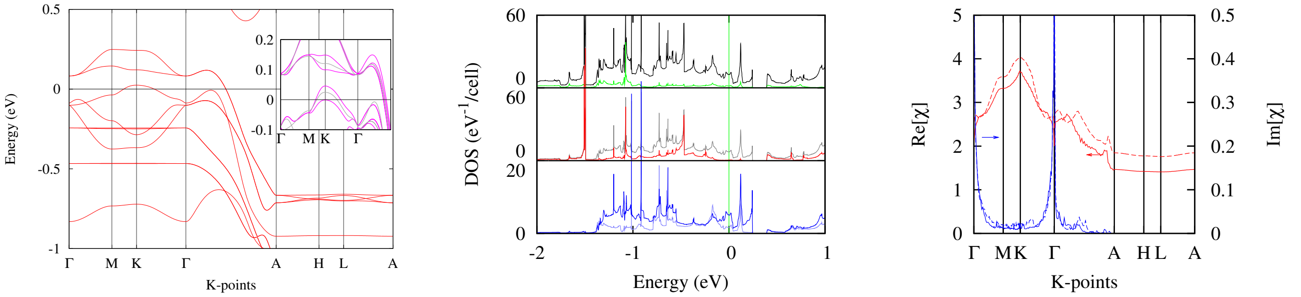

(a) (b) (c)

FIG. S-2: (a) Relativistic and non-relativistic band structure, (b) non-relativistic DOS and (c) bare electron susceptibility of $K_2Cr_3As_3$ with optimized lattice constants and internal coordinates. In panel a), the red lines show the non-relativistic result (without SOC effect) and the blue lines show the relativistic result (with SOC effect). In (b), upper panel shows the total DOS (black line) and PDOS from As-4p (green line); middle and bottom panel shows the PDOS from all five-orbitals of Cr-3d (grey lines in the middle panel), the PDOS of Cr-3d$_{zx}$ and Cr-3d$_{zy}$ (red line in the middle panel), and the PDOS of Cr-3d$_{z^2}$ (light blue line in the bottom panel), Cr-3d$_{x^2-y^2}$ and Cr-3d$_{xy}$(blue line in the bottom panel). In (c), The red and blue lines are the real and imaginary part of χ, respectively. The solid and dashed lines are results for prestine $K_2Cr_3As_3$ and 0.2 hole doped $K_2Cr_3As_3$, respectively.

We show here the band structure (FIG. S-2a) and DOS (FIG. S-2b) of $K_2Cr_3As_3$ with optimized lattice constants and internal coordinates. The most profound change in the band structure is that the γ band is submerged below E_F

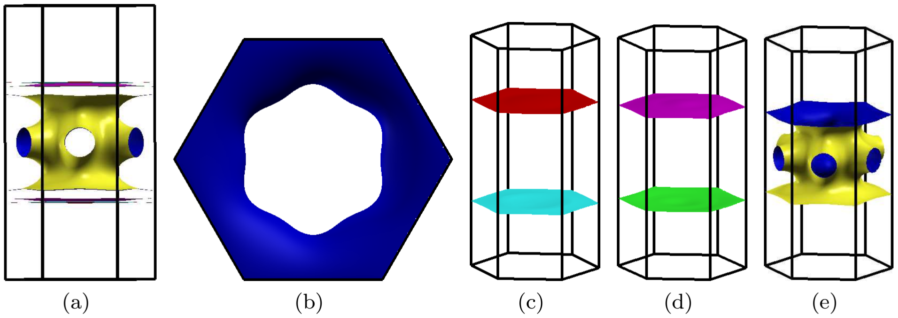

FIG. S-3: Non-relativistic Fermi surface sheets of $K_2Cr_3As_3$ with optimized lattice constants and internal coordinates. (a) Side-view of all sheets superposed. (b) Top view of 3D γ band sheet. (c-e) 3D view of α, β, and γ band sheets, respectively. The choice of high symmetry points is the same as FIG. 3c.

around Γ after structural relaxation. This will lead to a topologically different Fermi surface sheet (FIG. S-3(b-c)) from the one shown in FIG. 3(e-f). The α and β bands are less affected (FIG. S-3(c-d)). The number of bands crossing E_F, and their respective orbital character remains the same. Also, it is apparent that the SOC effect is large on γ and β, but is almost negligible for α (inset of FIG. S-2a).

The resulting non-relativistic band structure was also fitted to a 10-orbital tight-binding Hamiltonian using MLWF. Using this Hamiltonian, we have also calculated the bare electron susceptibility of both prestine $K_2Cr_3As_3$ and 0.2 hole doped $K_2Cr_3As_3$ (or, $K_{1.8}Cr_3As_3$) (FIG. S-2c). Despite of the detailed changes in the band structure, the prominent peak of Imχ_0 at Γ is insensitive to the structural change.

Electronic structure with idealized structure

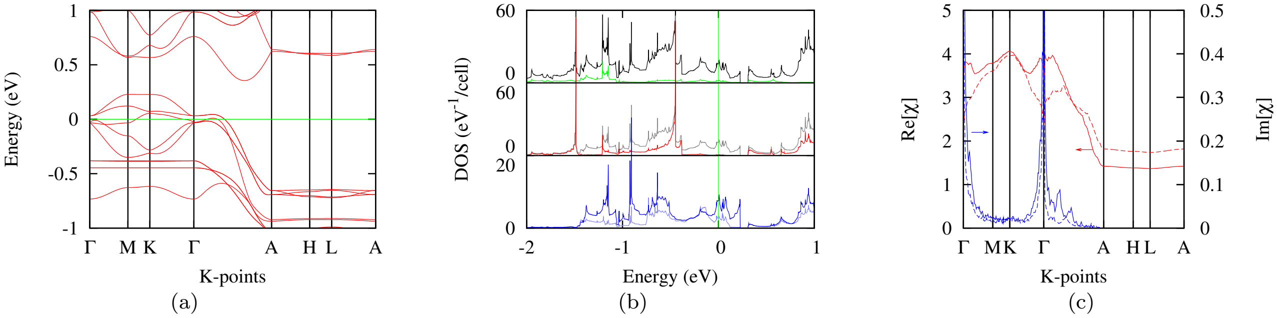

FIG. S-4: Non-relativistic (a) band structure (b) DOS and (c) bare electron susceptibility of $K_2Cr_3As_3$ with idealized structure. In panel (b), upper panel shows the total DOS (black line) and PDOS from As-4p (green line); middle and bottom panel shows the PDOS from all five-orbitals of Cr-3d (grey lines in the middle panel), the PDOS of Cr-3d$_{zx}$ and Cr-3d$_{zy}$ (red line in the middle panel), and the PDOS of Cr-3d$_{z^2}$ (light blue line in the bottom panel), Cr-3d$_{x^2-y^2}$ and Cr-3d$_{xy}$(blue line in the bottom panel). In panel (c), the red and blue lines are the real and imaginary part of χ, respectively. The solid and dashed lines are results for prestine $K_2Cr_3As_3$ and 0.2 hole doped $K_2Cr_3As_3$, respectively.

We have also calculated the nonrelativistic electronic structure of $K_2Cr_3As_3$ with an idealized structure, i.e. the Cr_6 cluster forms an ideal octahedron without distortion. We show here the band structure (FIG. S-4a), DOS (FIG. S-4b), bare electron susceptibility χ_0 (FIG. S-4c) and Fermi surface sheets (FIG. S-5). As indicated in these results, the number of bands crossing E_F, the respective orbital characters and dimensionality characters of these bands, the number of Fermi surface sheets and the prominent peak of Imχ_0 at Γ are insensitive to the structural change.

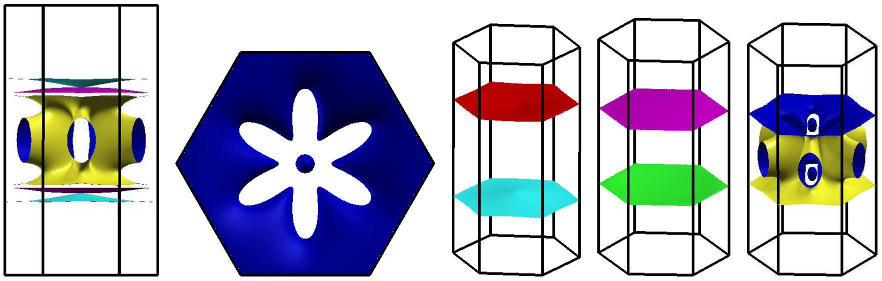

(a) (b) (c) (d) (e)

FIG. S-5: Non-relativistic Fermi surface sheets of $K_2Cr_3As_3$ with an idealized structure. (a) Side-view of all sheets superposed. (b) Top view of 3D γ band sheet. (c-e) 3D view of α, β, and γ band sheets, respectively. The choice of high symmetry points is the same as FIG. 3c.

Electronic structure at DFT+U level

Figure S-6 shows the electronic structure of $K_2Cr_3As_3$ under DFT+U with U=2.0 eV and J=0.7 eV. The band structure, DOS, Fermi surfaces and bare electron susceptibility are almost identical to those without U, suggesting that DFT+U is not very effective in the current system and will not affect our main conclusions.

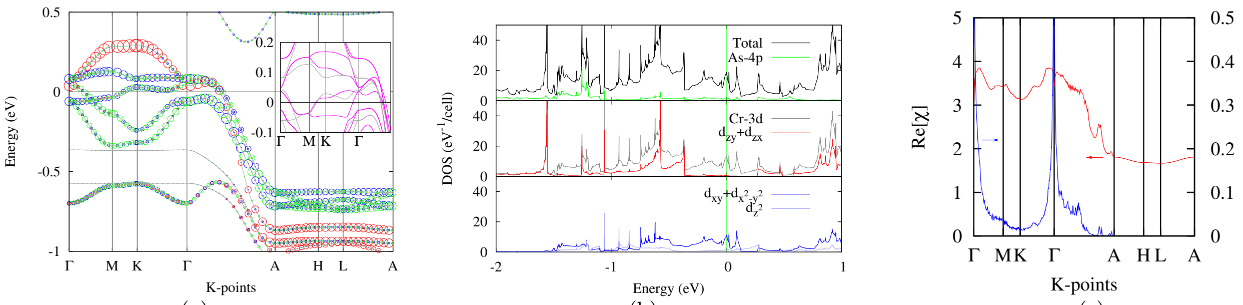

(a) (b) (c)

FIG. S-6: (a) Electronic band structure (b) Electron density of states and (c) Bare electron susceptibility of $K_2Cr_3As_3$ with experimental structure at U=2.0 eV and J=0.7 eV. In panel (a), the inset shows comparison between relativistic (magenta solid lines) and non-relativistic (grey solid lines) results. The size of the red, blue and green circles are proportional to the contributions from the d_{z^2}, $d_{x^2-y^2}$ and d_{xy} orbitals, respectively. In (b), the upper panel shows the total DOS (black line) and PDOS from As-4p (green line); middle and bottom panel shows the PDOS from all five-orbitals of Cr-3d (grey lines in the middle panel), the PDOS of Cr-3d_{zx} and Cr-3d_{zy} (red line in the middle panel), and the PDOS of Cr-3d_{z^2} (light blue line in the bottom panel), Cr-3$d_{x^2-y^2}$ and Cr-3d_{xy} (blue line in the bottom panel).
